# The lncRNAs involved in regulating the RIG-I signaling pathway

**DOI:** 10.3389/fcimb.2022.1041682

**Published:** 2022-11-09

**Authors:** Jing Liu, Qinglu Ji, Feng Cheng, Dengwang Chen, Tingting Geng, Yueyue Huang, Jidong Zhang, Yuqi He, Tao Song

**Affiliations:** ^1^ Department of Immunology, Zunyi Medical University, Zunyi, China; ^2^ School of Pharmacy, Zunyi Medical University, Zunyi, China; ^3^ Collaborative Innovation Center of Tissue Damage Repair and Regeneration Medicine, Zunyi Medical University, Zunyi, China; ^4^ Special Key Laboratory of Gene Detection and Therapy of Guizhou Province, Zunyi Medical University, Zunyi, China

**Keywords:** RIG-I, antiviral, antitumor, COVID-19, IFNs, lncRNAs

## Abstract

Understanding the targets and interactions of long non-coding RNAs (lncRNAs) related to the retinoic acid-inducible gene-I (RIG-I) signaling pathway is essential for developing interventions, which would enable directing the host inflammatory response regulation toward protective immunity. In the RIG-I signaling pathway, lncRNAs are involved in the important processes of ubiquitination, phosphorylation, and glycolysis, thus promoting the transport of the interferon regulatory factors 3 and 7 (IRF3 and IRF7) and the nuclear factor kappa B (NF-κB) into the nucleus, and activating recruitment of type I interferons (IFN-I) and inflammatory factors to the antiviral action site. In addition, the RIG-I signaling pathway has recently been reported to contain the targets of coronavirus disease-19 (COVID-19)-related lncRNAs. The molecules in the RIG-I signaling pathway are directly regulated by the lncRNA–microRNAs (miRNAs)–messenger RNA (mRNA) axis. Therefore, targeting this axis has become a novel strategy for the diagnosis and treatment of cancer. In this paper, the studies on the regulation of the RIG-I signaling pathway by lncRNAs during viral infections and cancer are comprehensively analyzed. The aim is to provide a solid foundation of information for conducting further detailed studies on lncRNAs and RIG-I in the future and also contribute to clinical drug development.

## Introduction

The innate immune system serves as the first line of defense against viral infection and is equipped with patterns recognition receptors (PRRs) (Kumar et al., 2011). The inflammatory cascade initiates with the recognition of microorganism-derived pathogen-associated molecular patterns (PAMPs) and host cell-derived damage-associated molecular patterns (DAMPs) by the PRRs (Gong et al., 2020; Li and Chang, 2021; Ragland and Kagan, 2021). This interaction activates the downstream signaling pathways and the consequent production of IFNs and pro-inflammatory cytokines to resist the pathogens infection directly and recruit the immune cells to eliminate the primary infection indirectly (Liu et al., 2016; Takaoka and Yamada, 2019).

**Figure 1:**
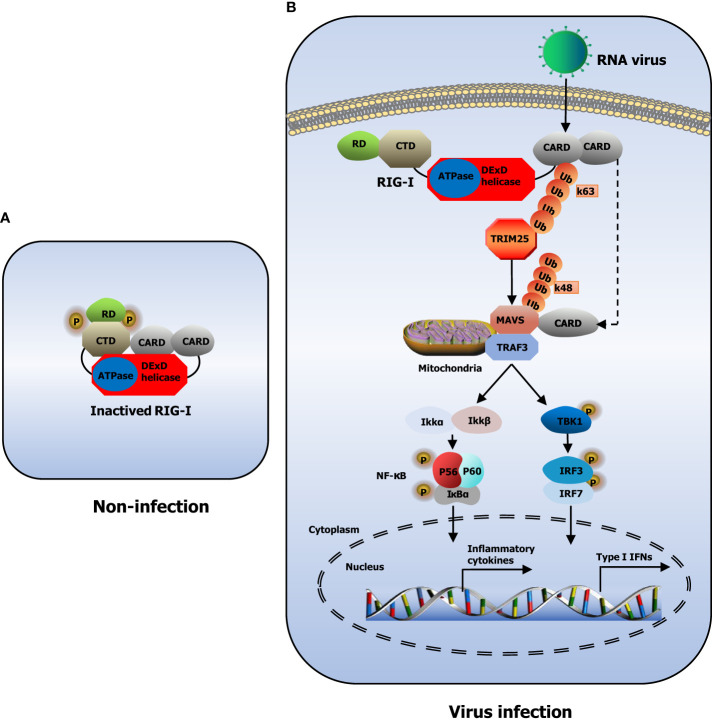
Model of RIG-I structure and signaling pathway. **(A)** The structure of RIG-I in non-infected cells. **(B)** RIG-I signaling pathway. During RNA virus infection, RIG-I recognizes RNA molecules and undergoes conformational changes that expose and multimerize its CARDs, which allows homotypic CARD–CARD interactions with MAVS. MAVS is anchored into mitochondria and relays the signal to TBK1 and IKK. These in turn activate IRF3 and IRF7, which together with the transcription factor NF-κB induce expression of type I IFNs and inflammatory cytokines.

PRRs in the innate immune system are broadly classified according to the homology of the protein domain in toll-like receptors (TLRs) (Kawai and Akira, 2010), RIG-I-like receptors (RLRs) (Wu et al., 2017), nucleotide oligomerization domain (NOD)-like receptors (NLRs) (Fekete et al., 2018), C-type lectin receptors (CLRs) (Willment, 2022), and absent in melanoma-2 (AIM2)-like receptors (ALRs) (Li and Wu, 2021). RLRs include RIG-I, melanoma differentiation-associated gene 5 (MDA5), and laboratory of genetics and physiology 2 (LGP2) (Uchikawa et al., 2016). RIG-I, also known as DExD/H-box helicase 58 (DDX58), belongs to the DExD/H box RNA helicase family of proteins (Banerjee et al., 2020). RIG-I contains a conserved ‘‘helicase’’ core, which comprises an N-terminus of two caspase activation and recruitment domains (CARDs), one DExD/H box RNA helicase domain, and a C-terminus of one C-terminal domain (CTD) and one repressor domain (RD) (Kowalinski et al., 2011). In non-infected cells, RD wraps the RNA-binding helicase domain while the CARDs are folded over one another, conferring an auto-repressed conformation to RIG-I in this state (Kageyama et al., 2011; Yong et al., 2019)**(** **Figure 1A** **)**. Upon viral infection, CARD in RIG-I is exposed, allowing itself to interact with tripartite motif-containing protein 25 (TRIM25) and mitochondrial antiviral signaling gene (MAVS) (Mu and Hur, 2021). CARDs of RIG-I interact with the TRIM25 E3 ligase, and this interaction effectively delivers the K63-linked ubiquitin moieties to the RIG-I second CARD, resulting in a marked increase of RIG-I downstream signaling activity (Gack et al., 2008). Furthermore, RIG-I is recruited to the adaptor protein MAVS located on the outer membranes of mitochondria after K63-linked ubiquitination (Gack et al., 2008). After binding, MAVS activates the tumor necrosis factor (TNF) receptor-associated factor 3 (TRAF3), which leads to the recruitment of tank-binding kinase 1 (TBK1) and inhibitor of kappa-B kinase (IKK) (Matthys et al., 2014). Subsequently, TBK1 and IKK phosphorylate the transcription factors IRF3 and IRF7, which then work synergistically with the transcription factor NF-κB to induce the expression of IFNs and proinflammatory cytokines (Taft et al., 2021; van der Wijst et al., 2021). The released IFNs and cytokines ultimately execute the antiviral action to eliminate the pathogen (Liu et al., 2017; Okamoto et al., 2017; Hou et al., 2021)**(** **Figure 1B** **)**.

The discovery and characterization of lncRNAs in recent decades have revealed that lncRNAs are larger transcripts (>200nt in size) without a protein-coding capacity. However, lncRNAs may have the splicing products of RNA polymerase II or III, 5’-capped, and 3’-polyadenylation (Wang et al., 2020). Research on novel prototype molecules has revealed that lncRNAs can be divided into four types according to their functions: signals, decoys, guides, and scaffolds (Wang and Chang, 2011; Ming et al., 2021). These lncRNAs are involved in the regulation of different processes, including genomic imprinting, chromatin remodeling, transcription, cell cycle, splicing, mRNA stabilization, and protein translation (Chen et al., 2018; Meng et al., 2018; Sallam et al., 2018; Sun et al., 2018; Rinn and Chang, 2020; Long et al., 2021). Various studies have reported that RIG-I recognizes and binds to lncRNAs to manifest nonspecific antiviral, antitumor, and other immunoprotective effects (RIG-I is a tumor suppressor and biomarker of IFN-α efficacy in HCC, 2014; Hou et al., 2021; Johnson et al., 2021; Li and Wu, 2021). According to a recent finding, the RIG-I signaling pathway contains the targets of certain lncRNAs in COVID-19 infection(Yang et al., 2021).

The present review aimed to summarize the relevant studies in the literature, which have explored how lncRNAs modulate the RIG-I signaling pathway by creating feedback or feedforward regulatory loops. Understanding host-virus and host-tumor interactions would help to develop efficient therapeutic strategies for infection control and maintenance of immune homeostasis, thus providing a foundation for future clinical research on medication and drug development.

## LncRNAs regulate RIG-I negatively or positively

RIG-I reportedly recognizes viral RNAs during a viral infection, leading to the induction of downstream signaling pathways (Oshiumi et al., 2016). Therefore, it is understood that the mechanism underlying the specificity of RIG-I and the activation of RIG-I must be meticulously tuned through various types of negative or positive regulations (Soonthornvacharin et al., 2017; Quicke et al., 2017).

Interestingly, certain lncRNAs that serve as negative regulators of innate immunity may be hijacked by viruses, leading to the inhibition of the antiviral response. According to the existing literature, the self-lncRNA lnc-Lsm3b serves as a molecular decoy and competes with viral RNA to bind to RIG-I monomers *via* GA-rich motifs, which are located on the incomplete pairing strand of long stem structure. This process was reported to occur during the late phase of vesicular stomatitis virus (VSV) infection and stabilize the intramolecular interaction between the CARDs and helicase domains. Furthermore, both 5’ppp-lnc-Lsm3b and 5’-OH-lnc-Lsm3b were reported to effectively suppress double-stranded RNA (dsRNA)-induced ATPase activity of RIG-I (Garcin, 2018; Jiang et al., 2018; Bird, 2018). Meanwhile, the RIG-I protein remained in an auto-repressed conformation, which prevented the oligomerization of RIG-I, ultimately leading to the inhibition of RIG-I activation (Jiang et al., 2018). Similar to lnc-Lsm3b, lncRNA negative regulator of antiviral signaling (lncATV) was also shown to serve as a potent negative regulator of full-length RIG-I, inhibiting the production of IFN-I and IFN-stimulated genes (ISGs) to prevent excessive activation of signaling pathways (Fan et al., 2019). Therefore, an antagonistic blockade of the involved pathogen-hijacked receptors could serve as a promising strategy for the control of infection and the associated immunopathology (Li et al., 2022).

The activity of RIG-I is also positively modulated by certain types of lncRNA. For example, the lncRNA named growth-arrest-specific transcript 5 (GAS5) reportedly activated the zeste enhancer homolog 2 (EZH2)-mediated enhancement of RIG-I (Le et al., 2021). Another significant lncRNA named nuclear paraspeckle assembly transcript 1 (NEAT1) was reported to activate the RIG-I-IRF7 signaling pathway and led to the in hibition of Hantaan virus (HTNV) infection. On the one hand, NEAT1 positively regulated and promoted the expressions of RIG-I and DEXD/H box helicase 60 (DDX60), which then acted synergistically to promote IFNs production (Ma et al., 2017). DDX60 is a DExH-box helicase and an ISG, which has been described to have a role as accessory protein involved in RLRs signaling (Goubau et al., 2015; Oshiumi et al., 2015). On the other hand, NEAT1 could bind to splicing factor proline and glutamine rich (SFPQ) to form paraspeckles, thereby relieving the transcriptional inhibitory effect of SFPQ on RIG-I and DDX60 (Ma et al., 2017). This then promotes the activation of downstream pathway signals and increases interleukin 8 (IL-8) transcription (Imamura et al., 2014). MiR-485 is associated with SFPQ by targeting the RIG-I mRNA for dampening the antiviral IFN-I response (Rehwinkel and Gack, 2020). However, no studies have confirmed a direct relationship between miR-485 and lncRNA NEAT1.

## LncRNA functions as a ceRNA to target MAVS in the RIG-I signaling pathway

MAVS transmits signals from RLRs after infection with RNA virus (Sharma et al., 2021). A study reported that lncRNAs regulate the innate immune response and influence viral replication during influenza A virus (IAV) H1N1 infection, and their interaction with miRNAs was vital to the pathogenesis of the disease (Wang et al., 2021). Another study reported that miRNAs regulate the RIG-I signaling pathway by inhibiting mRNA translation or promoting mRNA degradation during IAV infection (Wang et al., 2021). Increasing evidence indicates that MAVS antiviral-related lncRNA (MARL) is a key regulator of antiviral immunity in teleost fish (Chu et al., 2020). MARL functions as a competing endogenous RNA (ceRNA) for miR-122 to control the expression of MAVS, thus helping the viruses to evade the MAVS-mediated antiviral response and inhibiting Siniperca chuatsi rhabdovirus (SCRV) replication (Chu et al., 2020).

## LncRNAs affect TRIM25-mediated ubiquitination in the RIG-I signaling pathway

Several studies have suggested that the unanchored ubiquitin chain binds to RIG-I to induce downstream phosphorylation (Feng et al., 2013). Ubiquitination of RIG-I is one of the most prevalent protein posttranslational modifications, and the common types are the K63-linked and K48-linked polyubiquitination modifications (Jiang et al., 2012). RIG-I undergoes covalent K63-linked ubiquitination in both CARDs and CTD, and this post-translational modification relies on E3 ligases such as ring finger protein 135 (RNF135, also called RIPLET) and TRIM25 (Lai et al., 2019; Yang et al., 2021). TRIM25 directly catalyzes the K63-linked or K48- linked ubiquitination of RIG-I, thereby mediating antiviral innate immune responses (Chen et al., 2019; Choudhury et al., 2020). Structural studies have shown that K63-Ub_n_ by E3 ligase TRIM25 stabilizes the 2CARD and RD regions into a signaling-active, oligomeric, “lock-washer” conformation, in which the ubiquitin chains are bound to the outer rim of the 2CARD tetramer (Peisley et al., 2014; Jin et al., 2019). The K48-linked polyubiquitination, on the other hand, is mediated by a minimum of E3 ligases and facilitates the promotion of RIG-I degradation upon ubiquitination (Chen et al., 2013; Liu et al., 2017). Notably, upon K63-, K48-, or K29-linked ubiquitination, TRAF3 selectively regulates the expression of the type I interferons and pro-inflammatory cytokines *via* the RIG-I signaling pathway (Gao et al., 2021). Multiple cellular interaction-related proteins and lncRNAs have been demonstrated to modulate the TRIM25-mediated ubiquitination of RIG-I CARD (Lai et al., 2021).

For instance, lncNSPL blocked the interaction between RIG-I and E3 ligase TRIM25, thus inhibiting the TRIM25-mediated K63-linked RIG-I ubiquitination and limiting the production of downstream antiviral mediators in the late stage of IAV infection (Jiang et al., 2022). On the contrary, the cytoplasmic lncRNA AVAN could directly interact with the E3 ubiquitin ligase TRIM25 to enhance the TRIM25-mediated K63-linked ubiquitination of RIG-I, thus promoting activation of the RIG-I signaling pathway and consequently inducing the expression of type I interferons. AVAN is also present in the nucleus in addition to the cytoplasm of a cell. This nuclear AVAN positively regulates the transcription of forkhead box O3A (FOXO3a) by directly associating with its promoter, thereby promoting its expression and enhancing its neutrophil-activating function (Lai et al., 2021). Another confirmed lncRNA named lnc-zc3h7a also exhibits a ubiquitination mechanism similar to cytoplasmic AVAN. Furthermore, lnc-zc3h7a binds to both TRIM25 and RIG-I, thereby stabilizing the TRIM25–RIG-I interaction and facilitating TRIM25-mediated K63-linked ubiquitination of RIG-I (Lin et al., 2019). Similarly, lnc-Lsm3b is also associated with ubiquitination. It is worth mentioning that lnc-Lsm3b inactivates RIG-I innate function in the late stage of innate response (Jiang et al., 2018), while lnc-zc3h7a activates RIG-I at the early stage (Lin et al., 2019).

## LncRNAs participate in phosphorylation-mediated regulation of the RIG-I signaling pathway

Phosphorylation is an essential post-translational modification that prevents aberrant RIG-I signaling in non-infected cells (Gack et al., 2010). It is noteworthy that dephosphorylation is also important for the regulation of the RIG-I signaling pathway (Wies et al., 2013). Mechanistically, RIG-I activity is regulated by phosphorylation and dephosphorylation in its RD. Threonine at amino acid (aa) 770 and serine at aa 854 to 855 of RIG-I are phosphorylated by casein kinase II (CK2) in the resting state of the cell and dephosphorylated when cells are infected by RNA virus (Sun et al., 2011). RIG-I is considered a key receptor for the detection of viral RNA in the cytosol, as it induces IFN-mediated innate immune responses to limit viral replication after its interaction with MAVS (Sun et al., 2011; Chiang et al., 2014). The kinases TBK1 and IKK phosphorylate MAVS, which then recruits IRF3 and IRF7 through its phosphorylation-binding domain and promotes the phosphorylation of IRF3 by TBK1 (Liu et al., 2015; Fang et al., 2017). Furthermore, the phosphorylated MAVS recruits the canonical protein kinase IKK to activate NF-κB, thereby inducing the NF-κB signaling pathways and, ultimately, the expressions of inflammatory cytokines (Webb et al., 2019). NF-κB, which is composed of p50 and p65 molecules, is a transcription factor that is rapidly induced by the binding of the inhibitors of the NF-κB (IκB) family proteins (Zhang et al., 2017). MAVS interacts with downstream pathway components such as TRAF3, activating the protein IKK and leading to the phosphorylation of IκB. These ultimately result in activation of the NF-κB signaling pathway *via* phosphorylation of P65 (Zhang et al., 2017; Cohen and Strickson, 2017). The differentially expressed lncRNAs that cause a reduction in phosphorylation in the RIG-I signaling pathway are summarized in the present report.

Another lncRNA named lncRNA-GM binds a large fraction of glutathione S-transferase M1 (GSTM1) during viral infection. This binding of lncRNA-GM and GSTM1 blocks the interaction of GSTM1 with the kinase TBK1, thereby reducing the GSTM1-mediated S-glutathionylation, enhancing TBK1 activity, and promoting the production of the downstream mediators of antiviral response. After virus infection, lncRNA-GM levels rapidly decrease, leading to the promotion of GSTM1-mediated S-glutathionylation of the Cys637 residue in TBK1. This generates an inactive form of TBK1 that is incapable of inducing IRF3 phosphorylation and transcription activity. Consequently, the RIG-I downstream signaling pathway is attenuated while the replication and escape of viruses are promoted (Wang et al., 2020). The glutaredoxin/S-glutathione axis, in association with the NF-κB family of proteins, induces a pro-inflammatory response (Nolin et al., 2014). However, whether lncRNA-GM regulates the inflammatory NF-κB signaling through direct interaction with GSTM1 remains to be investigated. The lncRNA maintenance of meristems like1 (MaIL1), a positive regulator of IFN synthesis, is induced by lipopolysaccharide (LPS) and TLR3/RIG-I [poly(I:C)] and other PRR ligands (Aznaourova et al., 2020). Knockdown of MaIL1 blunted optineurin (OPTN) aggregation, TBK1-dependent IRF3 phosphorylation, and type I IFN expression (Aznaourova et al., 2020; Suarez et al., 2020). IRF3 phosphorylation is also strengthened by an IFN-inducible cytoplasmic lncLrrc55-As, which binds to phosphatase methylesterase 1 (PME-1). Mechanistically, lncLrrc55-As promotes the interaction between PME-1 and protein phosphatase 2A (PP2A), thereby enhancing IRF3 and IFN-I production (Zhou et al., 2019). Interestingly, the overexpression of lncATV could antagonize virus-stimulated phosphorylation of IRF3 as well as that of TBK1 and signal transducer and activator of transcription 1 (STAT1) (Fan et al., 2019). Consistently with this, lncRNA actin filament-associated protein 1 antisense RNA1 (AFAP1-AS1) may bind to the phosphorylated IRF7 protein and undergo phosphorylation modification, which leads to the activation of the RIG-I-like receptor pathway (Tang et al., 2018).

A multi-function lncRNA named RIG-I-dependent IAV-upregulated noncoding RNA (RDUR) reportedly enhances host antiviral immunity by positively regulating the phosphorylation of IRF3. On the other hand, virus-induced expression of RDUR prevents the host from a critical inflammation reaction, possibly through negative feedback control of both IκBα phosphorylation and NF-κB p65 phosphorylation (Chen et al., 2021). Nuclear retention of p65 is independent of the upstream activities IKK or IκB. Furthermore, the phosphorylation of p65 is considered an important hallmark of the NF-κB signaling pathway activity (Su et al., 2018). Furthermore, a differential expression of the lncRNA TCONS_00058367 could reduce phosphorylation of the transcription factor p65 (p-p65) through negative regulation in antisense gene promyelocytic leukemia (PML) in transmissible gastroenteritis virus (TGEV)-infected intestinal porcine epithelial cell line-J2 (IPEC-J2) cells (Ma et al., 2019).

## LncRNAs regulate the expression of ISGs *via* the RIG-I signaling pathway

IFNs are considered important in the therapeutic targeting of cellular systems as these molecules bind specific receptor complexes and mediate the expressions of numerous antiviral effectors, such as ISGs (Fan et al., 2020). IFNs are reported to activate the Janus kinase-signal transducers and activators of transcription (JAK-STAT) signaling pathway, leading to the production of ISGs. ISGs are powerful effector proteins that exert various functions during an innate immune response, including antiviral action (Ouyang et al., 2016; Tzeng et al., 2021). The first ISG to be reported is lnc-MxA, which inhibits the RIG-I signaling pathway by forming RNA-DNA triplexes at the IFN-β promoter. Furthermore, lnc-MxA interferes with the binding of IRF3 and NF-κB to the promoter, thereby reducing the activation of IFN-β transcription and downstream signaling pathways (Li et al., 2019). Another ISG is the IFN-stimulated lncRNA (lncRNA ISR). It is speculated that lncRNA ISR is induced by IFN-β during IAV infection and is regulated *via* the RIG-I-dependent signaling pathway involving NF-κB. However, it remains to be explored whether IRF3 and IRF7 are involved in the regulation of the lncRNA ISR in the RIG-I-dependent signaling (Pan et al., 2019). Moreover, lnc-ISG20 is also proposed to be an ISG similar to ISG20 and, therefore, IAV-induced lnc-ISG20 enhances ISG20 translation and regulates viral replication (Chai et al., 2018). A systematic screening study revealed that ISG20 also controls Zika virus (ZIKV) infection (Ding et al., 2021). Furthermore, lncBST2/BISPR, lncISG15, and their coding neighbors are also activated by IFNs and increase in the liver of patients infected with hepatitis C virus (HCV), where these positively regulate the ISGs BST2 and ISG15 (Barriocanal et al., 2014). lncRNA-155, after being transcribed, is hydrolyzed by an enzyme to form a 65-nt stem-loop precursor miRNA (pre-miR-155). Later, this pre-miR-155 is transported to the cytoplasm, where it is processed by another enzyme and further cleaved in its terminal loop to generate a 22-nt RNA (miR-155) (Elton et al., 2013). The induction of this miR-155 upon IAV infection causes an increased production of RIG-I/NF-κB dependent IFN-β and several ISGs through the regulation of protein tyrosine phosphatase 1B (PTP1B)-mediated interferon response (Maarouf et al., 2019).

Initial transcription of multiple ISGs is inhibited by the induction of lncATV, which then negatively regulates the RIG-I-mediated antiviral signaling and consequently prevents the excessive production of IFNs and inflammatory factors (Fan et al., 2019). Furthermore, the histone modifications of several key ISG genes, such as IFN-induced gene with tetratricopeptide repeats 2 (IFIT2), IFIT3, IFN-induced transmembrane protein 3 (IFITM3), and MxA, are inhibited upon negative regulator of antiviral ;response eplication (Ouyang et al., 2014). A similar phenomenon was also reported for the regulation of lncRNA cytidine monophosphate kinase 2 (CMPK2)/negative regulator of IFN response (NRIR)/eosinophil granule ontogeny transcript (EGOT), which suppresses the transcription of ISGs and inhibits the replication of HCV or influenza virus (Kambara et al., 2014; Carnero et al., 2016; Mariotti et al., 2019). LncRNA-IFI6, inducted by IFN-α, reportedly increased HCV replication by modulating the function of the ISG IFI6 promoter and histone modification independently of the JAK-STAT pathway (Liu et al., 2018). Moreover, in a PD rat model, a study has revealed that 512 differentially expressed lncRNAs (44 upregulated and 10 downregulated by recognized lncRNAs; 407 up-regulated and 51 down-regulated by novel lncRNAs) were associated with IRF7 and ISG15 in the RIG-I-like receptor signaling pathway (Li et al., 2019).

**Figure 2:**
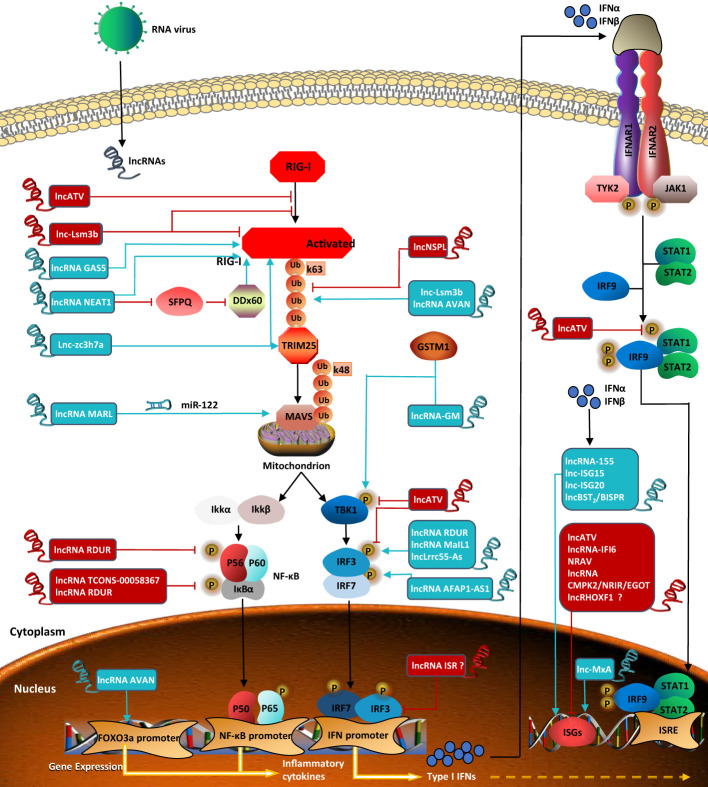
LncRNAs regulate RIG-I signaling pathway in viral infection. Lnc-Lsm3b inhibits the activation of RIG-I Lnc-Lsm3b inhibits the activation of RIG-I and affects ATPase. LncRNA GASS positively regulate RIG-I. LncRNA NEAT1 positively regulates the expression of RIG-I and DDX60. LncNSPL inhibits TRIM25-mediated RIG-I ubiquitination. Lnc-Lsm3b and lncRNA AVAN promote TRIM25-mediated polyubiquitination of RIG-I Lnczc3h7a binds to both TRIM25 and the RIG-I to facilitate TRIM25-mediated K63-linked ubiquitination of RIG-I. LncRNA MARL controls theexpression of MAVS by miR-122. LncRNA RDUR reduce the phosphorylation level of both p65 and I_k_b. LncRNA TCONS-00058367 reduces the phosphorylation level of transcription factor p65. LncRNA-GM induces TBK-1 phosphorylation. Lnc-ATV antagonizes virus-stimulated phosphorylations of TBK1 and IRF3. LncRNA RDUR, lncLrrc55-As, and lncRNAMaIL1 induce IRF3phosphorylation. LncRNA AFAP1-AS1 induces IRF7 phosphorylation. LncRNA AVAN positicely regulates the transcription of FOXO3a in the nucleus. LncRNA ISR and lnc-MxA are both ISGs. Lnc-MxA regulates RIG-I signaling pathway positively. LncRNA ISR negatively regulate IFNs-activated signaling, but it is unknown whether IRF3 and IRF7 are involved in the regulation of the expression. LncRNA-155, lnc-ISG15, lnc-ISG20 and lncBST2/BISPR activate ISGs transcription. Lnc-ATV, lncRNA-IFI6, NRAV, and lncRNA CMPK2/NRIR/EGOT suppress innate antiviral response by negatively regulating ISGs, but whether lncRHOXF1 directly regulates ISGs expression is unknown. LncRNA in blue represents positive regulators; lncRNA in red negative regulators for RIG-I signaling pathway.

While hundreds of ISGs have been identified so far, little is understood regarding IFN-stimulated lncRNAs. Interestingly, lncRHOXF1, which originates from the X chromosome and is expressed abundantly in trophectoderm and primitive endoderm cells of human blastocyst-stage embryos, increases the expression of viral response genes by using small interfering RNAs (siRNAs) during early human development. However, whether lncRHOXF1 directly activates IFN-β and/or ISGs needs further study (Penkala et al., 2016). LncRNAs, which are involved in the regulation of the RIG-I signaling pathway and described in the above sections, are all summarized in **Figure 2**.

## The triangle relationship between lncRNAs, RIG-I signaling pathway, and glycolysis

**Figure 3:**
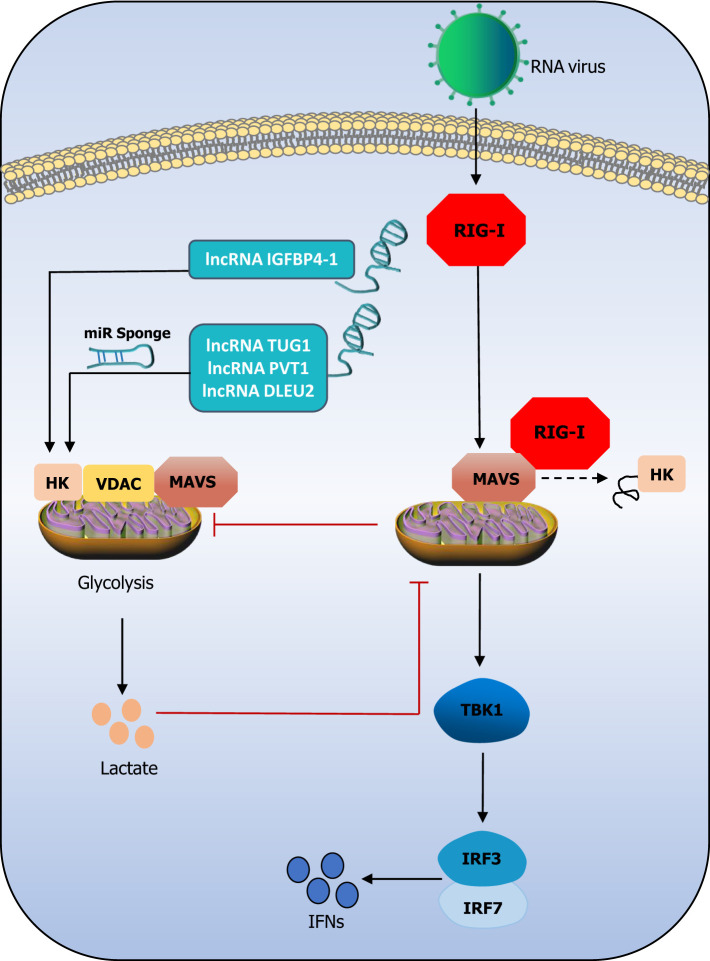
The triangle relationship between lncRNAs, the RIG-I signaling pathway, and glycolysis. LncRNAs promote HK activity abd lactate production. Lactate negatively regulates MAVS and subsequently impress TBK1-IRF3 activation and IFN-I production. Mitochondria HK activity inac-tivated, and glycolysis is suffered blocks during RIG-I activation. LncRNA IGFBP4–1 increases the expression of HK through their miR sponge. It could be that lncRNAs regulate HK and inhibit the binding of MAVS to RIG-I, thereby inhibiting the intensity of the RIG-I signaling pathway.

In addition to regulating multiple pathways involved in the antiviral innate immune response at various regulatory points, lncRNAs also regulate the glycolytic network (Liu et al., 2022). LncRNAs have been reported to cluster during the glycolytic cycle and inhibit viral replication through the disruption of hexokinase (HK2), voltage-dependent anion channel (VDAC), and MAVS ternary complex, thereby inhibiting glycolysis during viral infection (Ren et al., 2021). MAVS, the protein that is specific to signaling downstream of RIG-I, is reported to be the connecting link between antiviral immunity and glycolysis (Zhang et al., 2019). When MAVS switches its binding from HK2 to RIG-I, the result is the impairment of the mitochondrial localization and activity of HK2. On the other hand, lactate production inhibits the MAVS–RIG-I interaction, thus altering subsequent glycolysis and the downstream signaling pathways (Zhou et al., 2021). A previous study demonstrated that the lncRNA IGFBP4–1 increases the expression of enzymes, including HK (Yang et al., 2017). The lncRNA taurine upregulated 1 (TUG1) (Lin et al., 2018), plasmacytoma variant translocation 1 (PVT1) (Chen et al., 2019), and deleted in lymphocytic leukaemia 2 (DLEU2) (Dong et al., 2021) have been reported to positively regulate the expression of HK through their ceRNAs. While lncRNAs may regulate the HK-dependent lactate production by upstream kinase stimulation, MAVS is inhibited by HK and lactate (Ren et al., 2021). This exploration of the relationship among lncRNAs, HK, and RIG-I provides several insights. It could be that lncRNAs regulate HK and inhibit the binding of MAVS to RIG-I, thereby inhibiting the intensity of the RIG-I signaling pathway. However, these assumptions must be investigated and confirmed experimentally. **Figure 3** attempts to describe these assumptions. Recently, a study reported that severe acute respiratory syndrome coronavirus 2 (SARS-CoV-2)-induced monocyte immune response and viral replication require aerobic glycolysis support. An oxygen-sensing transcription factor named hypoxia-inducible factor-1α (HIF-1α) may induce glycolysis, leading to a pro-inflammatory response state in SARS-CoV-2-infected monocytes (Codo et al., 2020). The HIF-1alpha-stabilizing lncRNA (HISLA) inhibits the binding of HIF-1α to the prolyl hydroxylase domain-2 (PHD2) protein, which ensures the stability of HIF-1α and prevents its degradation, thereby maintaining the continuous activation of aerobic state *via* HIF-1α signaling and promotion of aerobic glycolysis (Chen et al., 2019). In general, HIF-1α acts as a bridge to connect lncRNA and glycolysis in SARS-CoV-2 infection. Otherwise, it was reported that the SARS-CoV-2 membrane (M) and nucleocapsid (N) protein could inhibit the production of type I and III IFNs by targeting RIG-I/MDA-5-MAVS signaling (Chen et al., 2020; Zheng et al., 2020; Zhao et al., 2021; Marx et al., 2022). Therefore, the above assumptions could also be applicable to SARS-CoV-2 infection, which is an adequate motivation to explore the relationship between lncRNAs, glycolysis, and the RIG-I signaling pathway.

## LncRNAs regulate the RIG-I signaling pathway in COVID-19 infection

The outbreak of 2019-nCoV demonstrated how important it was to identify novel virus-host interaction sites and virus escape mechanisms to develop further effective approaches to treat infectious diseases. It is worth noting that RIG-I recognizes the 3’-untranslated region of the SARS-CoV-2 RNA genome through its helicase domain, but not the C-terminal domain. And this new model of RIG-I recognition does not stimulate its ATPase (Yamada et al., 2021). In addition, lncRNAs are differentially expressed in terms of their levels in SARS-CoV-2 infection cells (Devadoss et al., 2021; Chattopadhyay et al., 2022). In contrast to the 3’-region of the SARS-CoV-2 genome, the 5’-region is capable of interacting with several human lncRNAs (Moazzam-Jazi et al., 2021). On the one hand, after SARS-CoV-2 infection, aerobic glycolysis increases, causing inflammation and even cytokine storms and resulting in increased viral replication (Thai et al., 2014; Ren et al., 2021; Zhu et al., 2021). On the other hand, SARS-CoV-2 nonstructural protein5 (nsp5) cleaves RIG-I and promotes MAVS degradation, which enables the virus to evade innate immunity (Liu et al., 2021; Zheng et al., 2022). Yang et al. summarized the significant lncRNAs associated with COVID-19 (Yang et al., 2021). When the ENCORI database was searched for any targets of these listed lncRNAs in the RIG-I signaling pathway, some targets were identified. The generated results are tabulated in **Table 1**. LncRNA EGOT is induced by NF-κB and functions as a negative regulator of the type I interferon response in SARS-CoV-2-infected patients (Mukherjee et al., 2021). LncRNA metastasis-associated lung adenocarcinoma transcript 1 (MALAT1)/wound and keratinocyte migration-associated long noncoding RNA 2 (WAKMAR2)/differentiation antagonizing non-protein coding RNA (DANCR)/NONHSAT122723.2 is highly likely to regulate lung inflammatory injury caused by cytokines through the NF-κB (Meydan et al., 2020; Mukherjee et al., 2021; Wu et al., 2021). LncRNA MALAT1/NEAT1 may regulate the RIG-I signaling pathway by targeting TRIM25 during SARS-CoV-2 infection from ENCORI database. Accumulating evidence indicates that lncRNAs are essential regulators of virus infections and antiviral immune responses by regulating the RIG-I signaling pathway, including biological processes that are involved in the regulation of COVID-19 and subsequent disease states (Yang et al., 2021). As stated earlier, the SARS-CoV-2 M and N protein represses the production of IFNs by interfering with the RIG-I signaling pathway, while immune evasion of the virus is related to IFN production. An improved understanding of the viral IFN antagonists involved in SARS-CoV-2 pathogenesis would have important implications for the development of new antiviral drugs and vaccines against this virus.

**Table 1:** Targets of lncRNAs in the RIG-I signaling pathway in COVID-19 infection. The targets we found in the ENCORI database are marked in red.

lncRNAs	Tagets	lncRNAs	Tagets
**LINC02384**	—	**NONHSAT122723.2**	NF-κB
**EGOT**	IFN-I, NF-κB	**Adapt33**	—
**MALAT1**	NF-κB, TRIM25	**Gm26917**	IFN-α
**NEAT1**	TRIM25	**SNHG1**	—
**AL392172**	—	**SMC2-AS1**	—
**HOTAIRM1**	—	**AC009088**	—
**PVT1**	—	**LINC02384**	—
**WAKMAR2**	NF-κB	**AL392172**	—
**EPB41L4A-AS1**	—	**HOTAIRM1**	—
**ENSG00000271646**	—	**PVT1**	—
**NORAD**	—	**SNHG25**	—
**RAD51-AS1**	—	**HIF1A-AS-1**	—
**GAS5**	—	**RORA-AS-7**	—
**DANCR**	NF-κB	**NRAV**	—
**NORAD**	—	**TUG1**	RIG-I
**RAD51-AS1**	—	**TTTY15**	—
**MSTRG.119845.30**	—	**TPTEP1**	—
**MSTRG.106112.2**	—	**GATA5**	—
**ENST00000631362**	—	**SNHG1**	—
**FTX**	—	**ROR1-AS1**	—

## LncRNAs exhibit antitumor immunity through the regulation of the RIG-I signaling pathway

A recent study summarized the relationship between typical cancers and lncRNAs, describing the importance of the latter as novel biomarkers and therapeutic targets in cancer (Mendell, 2016; Bhan et al., 2017; Chan and Tay, 2018). The most commonly reported lncRNA in this regard is the “miRNA sponge”, and this term was coined in 2011 by Salmena et al (Salmena et al., 2011). As the novel regulators of cancer-related signaling (Ma et al., 2018), lncRNAs act as elements that attach to miRNA competitively, and their differential expression may be exploited to identify potential candidates in different cancers and indirectly inhibit miRNA-targeted mRNA to regulate gene expression (Kwok et al., 2021). The lncRNA/miRNA/mRNA/ceRNA networks are crucial for antitumor immunity, as they regulate the RIG-I signaling pathway in correlation with the expression of human IFNs released from cancer cells (Liu et al., 2019; Calin, 2021). In the context of tumors, RIG-I signaling drives the transcriptional activation of a broad spectrum of pro-inflammatory genes, including those for IFN-I and pro-inflammatory cytokines (Bourquin et al., 2020; Iurescia et al., 2020; Boehmer et al., 2021). RIG-I exhibits tumor-suppressive or tumor-promoting (oncogenic) activity, depending on the different pathological stages of tumor progression (McCall et al., 2020). As in the case of SARS-CoV-2, human epithelial cancer cells are also reported to respond to cytosolic RNA *via* the RIG-I-MAVS-IRF3 pathway and sense cytosolic DNA *via* the cyclic GMP-AMP synthase (cGAS)-stimulator of interferon genes (STING) pathway. This was revealed by using cancer immunotherapy based on targeting nucleic acid receptors (Jena et al., 2020; Qiao et al., 2020; Liu et al., 2022).

Therefore, the investigation of the triad of lncRNA, RIG-I signaling pathway, and antitumor immunity becomes important. In this regard, RIG-I has been regarded as a functional downstream mediator of the lncRNA five prime to Xist (FTX)/miR-545 axis in hepatocellular carcinoma (HCC) cells (Liu et al., 2016). The overexpression of miR-545 could restore lncRNA FTX knockdown-induced inhibition, while the knockdown of miR-545 rescued the lncRNA FTX overexpression-induced promotion of HCC cell proliferation and cell cycle progression. The inhibition of miR-545 expression enhanced lncRNA FTX, which then suppressed the production of TNF-α, IL-6, IL-1β, and NF-κB (Liu et al., 2018).

**Figure 4:**
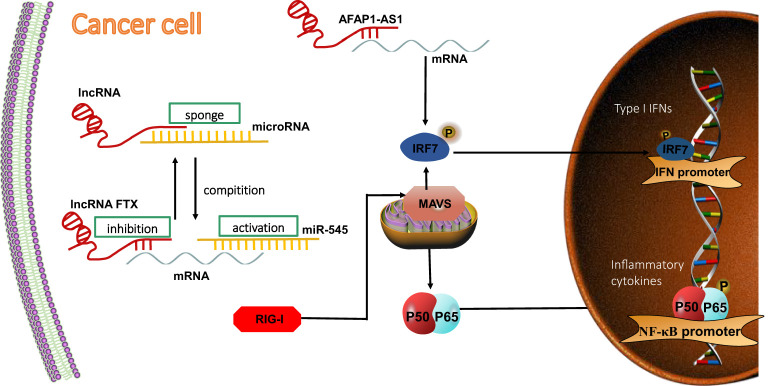
LncRNA regulates RIG-I signaling pathway in tumor cells. LncRNAs competitively bind to miRNA and indirectly inhibit miRNA-targeted mRNA expression, thereby inhibiting the activation of the RIG-I downstream signaling pathway. RIG-I acts as a direct target of miR-545. LncRNA FTX competitively binds to miR-545, thereby inhibiting mRNA expression in HCC cells. LncRNA AFAP1-AS1 affects mRNA, activating the positive-feedback regulation loop of IFN signaling and promoting migration and invation of NSCLC.

The RIG-I–lncRNA–tumor signaling axis is also regulated by IFN signaling. It has been reported that lncRNA AFAP1-AS1 is upregulated the most significantly in lung cancer and is also associated with poor prognosis (Zeng et al., 2016). Consistent with this, lncRNA AFAP1-AS1 could, through participation in the RIG-I signaling pathway, affect the expression of mRNA and protein of related genes, such as the one for IRF7, that are used as target genes in cancer therapy. This would activate the positive feedback regulation loop of IFN signaling and consequently promote the migration and invasion of non-small cell lung cancer (NSCLC) (Tang et al., 2018)**(** **Figure 4** **)**.

We found only two studies that reported the regulation of the RIG-I signaling pathway by lncRNAs during antitumor immunity. Anyway, with antitumor innate immune responses, several intriguing and crucial aspects remain to be investigated, and the results of such an investigation would further expand the understanding of the mechanism underlying the interaction between RIG-I and lncRNAs. The most recent studies have reported effective combination therapy techniques for metastatic and low-immunogenicity tumors (Domankevich et al., 2020; Peng et al., 2022). Future research may offer further promising choices for controlling the infection and the associated immunopathology.

## Conclusions and prospects

The increasing number of studies on the recognition of PRRs and their ligands in recent years have greatly improved our understanding of the RIG-I signaling pathway machinery and provided novel insights into the treatment of immune-related diseases (Hwang et al., 2022; You et al., 2022). With this, lncRNAs have emerged as a research hotspot in the field of medicine. Due to their crucial regulatory functions, lncRNAs are involved in a variety of biological processes and pathways that are closely related to the occurrence and development of multiple diseases (Kopp and Mendell, 2018).

In general, lncRNAs have been reported to modulate the host immune response by regulating the expression of RIG-I and MAVS, the production of IFNs and inflammatory factors, the transcription of antiviral ISGs, and a series of post-translational modifications, such as polyubiquitination, glycolysis, and phosphorylation. The sources of lncRNAs, which are associated with the regulation of the RIG-I signaling pathway, are listed in **Table 2**. However, the knowledge regarding the lncRNAs expressed by viruses is at the stage of infancy currently (Li et al., 2022). Therefore, the exploration of new viral lncRNAs and their mechanisms of regulating the RIG-I signaling pathway warrants further investigation. Despite the accumulation of information on lncRNAs, further research on lncRNAs is necessary to support the clinical diagnosis and meet the advancements in medical technology, as well as the current demand for drug development in the field of medicine.

**Table 2:** Sources of lncRNAs in the RIG-I signaling pathway.

LncRNA name	Sources of lncRNAs	Reference
**LncRNAs regulate RIG-I negatively or positively**		
**Lnc-Lsm3b**	Macrophages, multiple types of immune cells and organs	(Jiang et al. 2018)
**LncATV**	Monocytes, erythroleukemia cells and hepatoma cell	(Fan et al., 2019)
**LncRNA GAS5**	SMC and aortic tissue of AAA mice	(Le et al., 2021)
**LncRNA NEAT1**	T lymphocytic cell lines Jurkat, MT4 and THP-1 cells	(Ma et al. 2017)
**LncRNA functions as a ceRNA to target MAVS in the RIG-I signaling pathway**		
**LncRNA MARL**	Miiuy croaker chromosome 14	(Chu et al., 2020)
**LncRNAs affect TRIM25-mediated ubiquitination in the RIG-I signaling pathway**		
**LncNSPL**	IAV-infected THP-1 human monocytes	(Jiang et al., 2022)
**LncRNA AVAN**	PBMC, CD14’monocytes, derived macrophages, and BEAS-2B	(Lai et al. 2021)
**Lnc-Lsm3b**	Macrophages, multiple types of immune cells and organs	(Jiang et al. 2018)
**Lnc-zc3 h7a**	HEK 293T cells and RAW264.7 cells	(Lin et al. 2019)
**LncRNAs participate in phosphorylation-mediated regulation of the RIG-I signaling pathway**		
**LncRNA-GM**	Monocytes, macrophages and other immune cells, including CD4^+^T cells, CD8^+^T cells, γδT cells, NK cells, neutrophils, and eosinophils	(Wang et al., 2020)
**LncRNA MaIL1**	Macrophages and monocytes	(Aznaourova et al., 2020)
**LncLrrc55-As**	The antisense transcripts of the Lrrc55 gene	(Zhou et al., 2019)
**LncATV**	Monocytes, erythroleukemia cells and hepatoma cell	(Fan et al., 2019)
**LncRNA AFAP1-AS1**	Various tumor tissues and NSCLC cells lines	(Tang et al. 2018)
**LncRNA RDUR**	The cytoplasm and nucleus of A549cells	(Chen et al. 2021)
**LncRNA TCONS-00058367**	Physical contiguity PML	(Ma et al. 2019)
**LncRNAs regulate the expression of ISGs via the RIG-I signaling pathway**		
**Lnc-MxA**	Be an ISG in the nucleus in IAV-infected cells	(Li et al. 2019)
**LncRNA ISR**	A549 and HEK293T cells	(Pan et al. 2019)
**Lnc-ISG20**	A549 and HEK293T cells	(Chai et al. 2018)
**Lnc-ISG15**	HuH7 and A549 cells	(Barriocanal et al., 2014)
**LncBST2/BISPR**	HuH7 and A549 cells	(Barriocanal et al., 2014)
**LncRNA-155**	A549 cells	(Maarouf et al. 2019)
**LncRNA NRAV**	Chromosome 12q24.31 in A549 and 293T cell lines	(Ouyang et al. 2014)
**LncRNA CMPK2/NRIR/EGOT**	Human hepatocytes	(Carnero et al. 2016)
**LncRNA-IFI6**	JFH1-infected Huh7.5.1 cells and PHHs	(Liu et al., 2018)
**LncRHOXF1**	Human X chromosome	(Penkala et al. 2016)
**The triangle relationship between lncRNAs, RIG-I signaling pathway, and glycolysis**		
**LncRNA IGFBP4–1**	Human lung adenocarcinoma cancer cell lines (A549, PC-9, and GLC-82)	(Yang et al., 2017)
**LncRNA TUG1**	Hepatocellular carcinoma cell	(Lin et al., 2018)
**LncRNA PVT1**	GBC tissue	(Chen et al., 2019)
**LncRNA DLRU2**	HEC-1, HEC-50, HHUA, Ishikawa, KLE cells, and endometrial epithelial cell line	(Dong et al., 2021)
**LncRNA HISLA**	Macrophages	(Chen et al., 2019)
**LncRNAs regulate the RIG-I signaling pathway in COVID-19 infection**		
**LncRNA EGOT**	BALF	(Yang et al., 2021)
**LncRNA MALAT1**	BAL cells	
**LncRNA NEAT1**	NHBE cells, BAL cells	
**LncRNA WAKMAR2**	BALF	
**LncRNA DANCR**	Inflammation-prone lung tissues	
**LncRNA NONHSAT122723.2**	PBMC	
**LncRNA Gm26917**	Unknown	
**LncRNA TUG1**	Hepatocellular carcinoma cell	(Lin et al., 2018)
**LncRNAs exhibit antitumor immunity through the regulation of the RIG-I signaling pathway**		
**LncRNA AFAP1-AS1**	Various tumor tissues and NSCLC cells lines	(Tang et al. 2018)
**LncRNA FTX**	The cis-acting regulatory region of the imprinted XCI	(Liu et al., 2018)

The table summarizes the sources of different lncRNAs and references to the source articles. SMC, smooth muscle cells; AAA, Abdominal aortic aneurysm; PBMC, peripheral blood mononuclear cells; A549, human lung epithelial cells; HEK293T, human embryonic kidney cells; PML, promyelocytic leukemia; BEAS-2B, a human normal lung epithelial cell; GBC, Gallbladder cancer; NSCLC cell lines: A549, H1975, H1650, H1395, and H1299; PHHS, Primary Human Hepatocytes; Lrrc55, leucine rich repeat containing 55; BALF, bronchoalveolar lavage fluid; NHBE, normal human bronchial epithelial; XCI, X-chromosome inactivation.

A better understanding of the mechanisms observed for SARS-CoV-2-infected cells and the mediators involved in immune responses to this virus is a prerequisite for the development of diagnostic markers and therapeutic strategies targeting COVID-19. In this regard, a detailed exploration of the new targets of SARS-CoV-2-associated lncRNAs that are capable of binding to RIG-I would be the current challenge. It is worth noting that glycolysis could be a breakthrough.

Drug resistance is a well-recognized challenge encountered in the clinical treatment of various malignant tumors. In this sense, RIG-I participates in the innate immune response and is also involved in the regulation of apoptosis, which has been related to the occurrence and development of various tumors (Zander et al., 2022). The lncRNAs, referred to as “miRNA sponges”, downregulate the expression and activity of several miRNAs and subsequently modulate the de-repression of miRNA targets at the level of antitumor immunity regulation (Ghafouri-Fard et al., 2021). However, studies on the relationship between lncRNAs, the RIG-I signaling pathway, and antitumor immunity are scarce, and understanding this relationship would provide deeper insights into this field. Therefore, studying the mechanism of drug resistance in tumor cells and reversing the symptoms of drug resistance are considered major factors responsible for the successful drug treatment of patients. However, although the mechanism of tumor resistance has been widely discussed, it has not been fully understood so far.

This article provides a summary of research on the regulation of RIG-I by lncRNAs, which would be of assistance to scholars involved in this field. Future studies would further expand the repertoire of lncRNAs involved in the RIG-I regulatory circuit, thus providing critical information for potential therapeutic purposes. The identification of novel molecular targets for lncRNAs is imperative for the development of effective therapeutic methods to improve the prognosis of patients.

## Author contributions

JL conceived the idea, analysis of literature, and writing of the manuscript; QJ, FC, and DC performed manuscript preparation contributed collection; TG and YYH helped perform constructive discussions; JZ, YQH, and TS contributed to the conception of the review. All authors contributed to the article and approved the submitted version.

## Funding

This work was funded by the Program of the Guizhou Science and Technology Department, China (No. QKHPTRC[2018]5772-035); the Program of the Health Commission of Guizhou Province, China (No. GZWKJ-2021-533); the National Nature Science Foundation of China (NSFC) (No. 31960156, 31660338); Collaborative Innovation Center of Chinese Ministry of Education (2020-39); Guizhou University Dendrobium nobile industry development key technology engineering research center (No. QKJ-2022-048), Guizhou Provincial Department of Education "four new" and "four modernizations" science and technology research project (No. QJJ-2022-006).

## Conflict of interest

The authors declare that the research was conducted in the absence of any commercial or financial relationships that could be construed as a potential conflict of interest.

## Publisher’s note

All claims expressed in this article are solely those of the authors and do not necessarily represent those of their affiliated organizations, or those of the publisher, the editors and the reviewers. Any product that may be evaluated in this article, or claim that may be made by its manufacturer, is not guaranteed or endorsed by the publisher.
